# Twenty-Eight Years of Poliovirus Replication in an Immunodeficient Individual: Impact on the Global Polio Eradication Initiative

**DOI:** 10.1371/journal.ppat.1005114

**Published:** 2015-08-27

**Authors:** Glynis Dunn, Dimitra Klapsa, Thomas Wilton, Lindsay Stone, Philip D. Minor, Javier Martin

**Affiliations:** Division of Virology, National Institute for Biological Standards and Control, Potters Bar, Hertfordshire, United Kingdom; Columbia University, UNITED STATES

## Abstract

There are currently huge efforts by the World Health Organization and partners to complete global polio eradication. With the significant decline in poliomyelitis cases due to wild poliovirus in recent years, rare cases related to the use of live-attenuated oral polio vaccine assume greater importance. Poliovirus strains in the oral vaccine are known to quickly revert to neurovirulent phenotype following replication in humans after immunisation. These strains can transmit from person to person leading to poliomyelitis outbreaks and can replicate for long periods of time in immunodeficient individuals leading to paralysis or chronic infection, with currently no effective treatment to stop excretion from these patients. Here, we describe an individual who has been excreting type 2 vaccine-derived poliovirus for twenty eight years as estimated by the molecular clock established with VP1 capsid gene nucleotide sequences of serial isolates. This represents by far the longest period of excretion described from such a patient who is the only identified individual known to be excreting highly evolved vaccine-derived poliovirus at present. Using a range of *in vivo* and *in vitro* assays we show that the viruses are very virulent, antigenically drifted and excreted at high titre suggesting that such chronic excreters pose an obvious risk to the eradication programme. Our results in virus neutralization assays with human sera and immunisation-challenge experiments using transgenic mice expressing the human poliovirus receptor indicate that while maintaining high immunisation coverage will likely confer protection against paralytic disease caused by these viruses, significant changes in immunisation strategies might be required to effectively stop their occurrence and potential widespread transmission. Eventually, new stable live-attenuated polio vaccines with no risk of reversion might be required to respond to any poliovirus isolation in the post-eradication era.

## Author Summary

The global polio eradication initiative is the most ambitious and complex public health programme directed at a single disease in history with a projected cost of $16.5 billion. Of the three serotypes types 2 and 3 appear to have been eradicated in the wild and type 1 is mostly confined to a region of Pakistan and Afghanistan. There is a real probability of total eradication in the near future. The main vaccine used is a live attenuated virus, and our paper concerns one of the most intractable significant implications that this has for the polio endgame. We describe virological studies of a patient deficient in humoral immunity who has been excreting type 2 vaccine-derived poliovirus for 28 years. Our results show that the viruses are excreted at high titres, extremely virulent and antigenically drifted and raise questions about how the population may best be protected from them, particularly in the light of possible changes in vaccine production which are being encouraged to increase capability and reduce costs. The study has implications for the ecology of poliovirus in the human gut and highlights the risks that such vaccine-derived isolates pose for polio re-emergence in the post-eradication era.

## Introduction

Despite difficulties in interrupting wild poliovirus transmission in the last few remaining endemic countries and recent drawbacks due to international spread of poliovirus in central Asia, central Africa and the Middle East [1], the global polio eradication appears to be within reach. Four of the six WHO regions have been certified polio-free and a country such as India, where massive poliomyelitis outbreaks were very common, interrupted circulation of endemic wild poliovirus in 2010. There has been no case of poliomyelitis caused by circulating wild type 2 poliovirus since 1999, no case of type 3 since November 2012 and the last case of type 1 in Africa was in August 2014, leaving some areas of Pakistan and Afghanistan as the main remaining reservoirs [2]. All type 2 poliomyelitis cases since 1999, except an isolated incident of 10 cases linked to a wild laboratory reference strain in India [3], are due to vaccine-related poliovirus strains in either recipients, their immediate contacts or after the vaccine virus has regained the ability to transmit and circulate freely. Vaccine-associated paralytic poliomyelitis occurs in a very small proportion of vaccinees [4] and can be prevented by using inactivated rather than live vaccine. Vaccine-derived poliovirus (VDPV) strains, defined as those with more than 1% (0.6% for serotype 2 poliovirus) sequence drift in the capsid VP1 gene with respect to the corresponding Sabin strain, can be generated and transmitted from person to person in populations with low immunity and have been associated with a number of poliomyelitis outbreaks around the world [5–9]. These circulating VDPVs (cVDPVs) behave very similarly to wild polioviruses and should therefore be eliminated by the same immunisation methods. In addition, some hypogammaglobulinaemic patients are known to excrete poliovirus for prolonged periods of time [10–12] but there is currently no effective strategy to deal with this problem. Although there has been some evidence of local virus transmission from these patients to unvaccinated children [13], VDPV strains from immunodeficient individuals (iVDPVs) have not yet been implicated in outbreaks in the same way that cVDPVs have [14]. The World Health Organization (WHO) and partners have prepared endgame plans for the global polio eradication initiative (GPEI) which include the elimination of the serotype 2 component from the Sabin live-attenuated oral poliovaccine (OPV) and the implementation of global use of inactivated poliovaccine (IPV) [15]. This represents a major change after more than 50 years of trivalent OPV use for routine immunisation although monovalent and bivalent vaccines are commonly used for campaigns on national immunisation days.

The risks posed by iVDPV strains and the prevalence of such cases globally are unknown so their relevance in the context of the GPEI endgame is not easy to assess. In order to better understand the growth and properties of iVDPV strains and their potential for transmission, we have characterised iVDPV isolates from an immunodeficient individual obtained during a period of more than 20 years. Although examples of long-term poliovirus excretion have been described before by us and others (reviewed in [14]), they have mostly included a small number of samples from paralytic cases as otherwise asymptomatic long-term excreters remain undetected. In previous cases patients died, stopped shedding virus or were lost to follow up relatively soon after the first virus isolation. Important gaps in the scientific knowledge of long-term poliovirus excretion by these individuals remained such as determining changes in excretion titres, antigenic structure and neurovirulence of poliovirus following many years of evolution in a single individual as well as estimating the efficacy of current vaccines at preventing paralysis and transmission induced by these viruses. Our paper provides relevant findings in these areas that indicate that VDPV isolates form these patients represent a real risk of polio re-emergence in the post-eradication era, particularly considering there is currently no effective strategy to treat these patients.

## Results and Discussion

**Fig 1:**
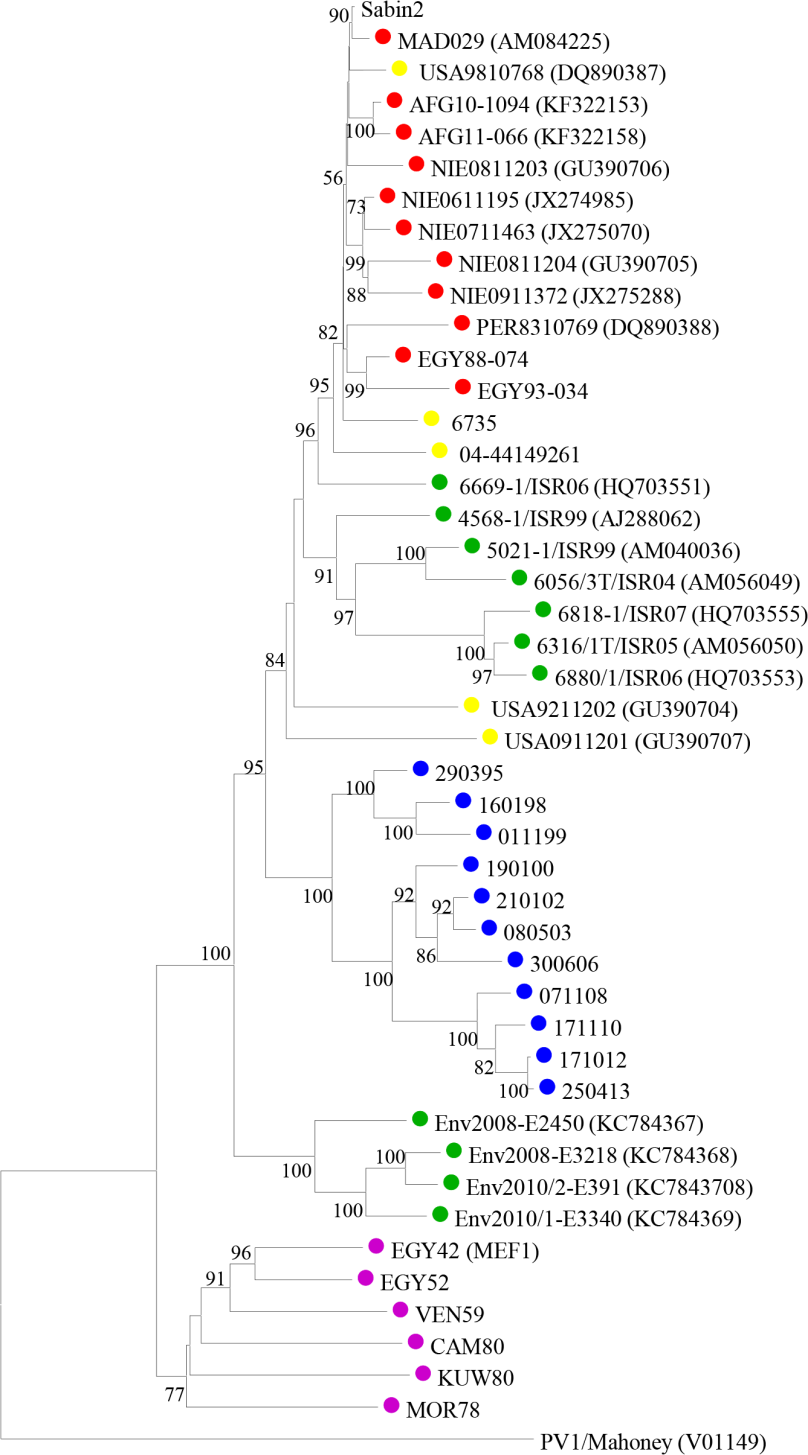
Sequence analysis of iVDPV strains. Neighbour-joining tree representing phylogenetic relationships through the entire capsid coding sequence (2637 nt) between iVDPV isolates from the case study (shown as a number that corresponds to the date of isolation in the format *ddmmyy*), Sabin 2 vaccine strain and other type 2 VDPV and wild polioviruses. EMBL Data Library accession numbers for published capsid sequences are shown in the tree. Numbers at nodes indicate the percentage of 1000 bootstrap pseudoreplicates supporting the cluster. The sequence of PV1-Mahoney reference strain was introduced as an outgroup for the correct rooting of the tree. Isolates from the patient are labelled with blue circles on the tree, other iVDPV isolates are indicated in yellow, cVDPVs in red, VDPVs found in sewage samples in green and wild polioviruses in purple.

The first stool samples from this individual were tested between March and November 1995. At that time, type 2 VDPV isolates differing from the parental Sabin 2 OPV strain at between 9.9% and 11.3% of VP1 nucleotides were identified. A total of 185 subsequent samples have been obtained so far in the following years, all positive for iVDPV2 strains with virus titres shed in the stools typically around or above 4 log_10_ infectious particles per gram, comparable to virus titres shed by healthy vaccinees and paralytic cases infected with vaccine or wild poliovirus [16]. The latest isolate available was from 4^th^ March 2015 showing a 17.7% VP1 sequence drift from Sabin 2 poliovirus. Phylogenetic analyses in the capsid region confirmed that the iVDPV strains were genetically related, sequentially evolved from Sabin 2 and distinct from other type 2 VDPVs and wild polioviruses (Fig 1).

A Bayesian Monte Carlo Markov Chain (MCMC) phylogenetic analysis determined a mean evolutionary rate of 1.51×10^−2^ total substitutions/site/year [95% High Probability Distribution (HPD_95_) range = 1.26–1.77×10^−2^] in the VP1 gene, similar to previous estimates for poliovirus VP1 [17]. The date of the initiating OPV dose was estimated to be 11^th^ March 1986 [HPD_95_ = 6^th^ July 1983-11^th^ January 1989], relatively close to 4^th^ August 1986, the date of the patient’s last known OPV vaccination. It is therefore most likely that this individual has been excreting poliovirus for around 28 years. There was no apparent effect on the virus evolution rate suggesting bottleneck effects due to the anti-viral treatments that failed to interrupt virus excretion from this patient [18]. However, a much more detailed analysis of virus population dynamics should be conducted to determine any possible effect due to the different anti-viral interventions.

**Fig 2:**
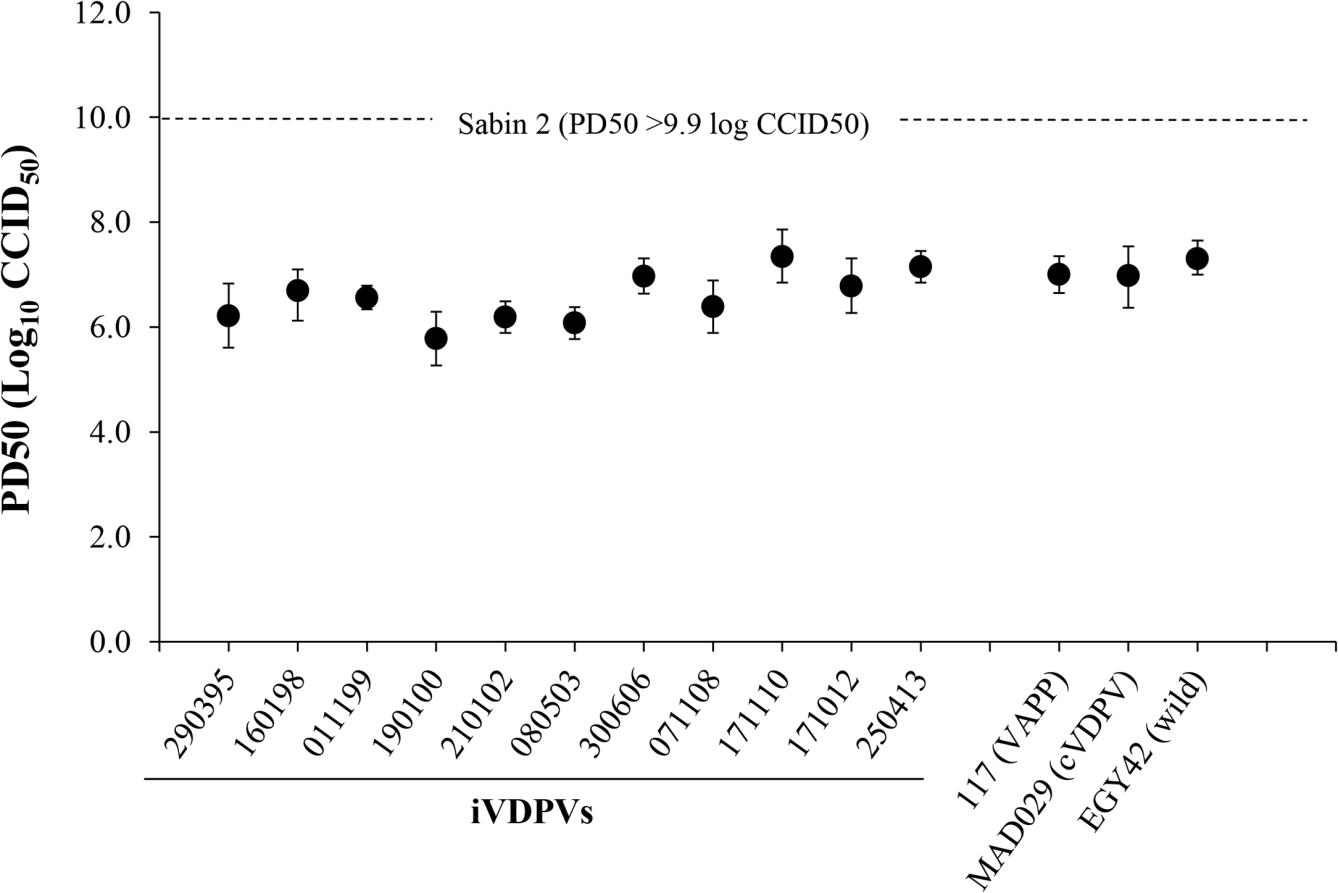
Neurovirulence of iVDPV strains. The graphic represents 50% Paralytic Doses (PD_50_) values (with 95% Confidence Intervals) for selected poliovirus strains determined in Tg21-bx mice using the Probit method. Isolates from the patient are shown underlined.

All iVDPV isolates showed reversion at the two known attenuation mutations of Sabin 2 vaccine strain: nucleotide 481 (from A to G) in the 5’ non-coding region (5’NCR) and capsid amino acid VP1-143 (from Isoleucine to Threonine) and were highly neurovirulent in transgenic mice expressing the human poliovirus receptor. The 50% paralytic dose (PD50) values were comparable to those determined for cVDPV and wild polioviruses while the Sabin 2 vaccine strain did not paralyse any animals at the highest dose that could be given (Fig 2).

Amino acid differences with respect to the Sabin 2 parental strain in the complete coding sequence of selected iVDPV isolates were determined (S1 Table). All iVDPV strains contained identical changes from Sabin 2 at 52 amino acid positions. Forty mutations were present in at least two iVDPV isolates and 24 amino acid changes were unique. The proportion of nucleotide mutations leading to amino acid changes was high for all iVDPV strains. This contrasts with the low proportion of non-synonymous changes from Sabin 2 identified in cVDPV strains and wild isolates as found here and elsewhere, particularly in capsid sequences. It is not clear whether any of the numerous additional mutations incorporated in the iVDPV isolates have any effect on neurovirulence but they do not seem to have an overall mitigation impact for any of the isolates tested as it has been reported for one highly drifted type 2 VDPV isolate found in a sewage sample in Israel [19]. Many of the sequence changes between the iVDPV strains and the Sabin 2 virus resulted in amino acid differences in known antigenic sites [20] (Table 1).

**Table 1:** Amino acid differences in antigenic sites between type 2 polioviruses.

	Antigenic Site																													
	Site 1 (VP1)						Site 2a (VP1)							Site 2b (VP2)				Site 3a (VP2)				Site 3b (VP3)								
Virus^a^	98	99	100	101	102	103	218	219	220	221	222	223	224	167	168	170	172	72	157	158	240	58	59	60	61	62	66	73	75	209
**Sabin 2**	T^b^	K	R	A	S	R	A	G	Q	A	S	T	E	T	N	A	N	R	K	G	T	T	S	Q	R	K	D	S	T	D
**160198 (i)**	-	N	-	T	-	K	-	S	H	-	A	-	D	-	-	-	E	N	E	R	-	-	-	-	H	R	-	T	A	V
**190100(i)**	-	N	-	T	-	K	-	-	H	-	A	-	D	-	-	-	K	N	-	-	-	-	-	-	H	R	-	T	A	V
**080503(i)**	-	N	-	T	-	K	-	-	H	-	A	A	D	-	S	-	K	N	-	-	-	-	-	-	H	R	-	T	A	V
**071108(i)**	-	N	-	-	-	K	-	-	H	-	A	A	D	-	-	-	K	N	-	-	A	-	-	-	H	R	-	T	A	V
**171012(i)**	-	N	-	-	-	K	-	-	H	T	A	*d*	D	-	S	-	K	N	-	-	A	-	-	-	H	R	-	T	A	V
**USA92(i)**	-	R	-	-	-	K	E	-	-	T	A	-	-	-	-	E	K	S	-	-	-	-	N	G	-	R	-	-	S	V
**USA09(i)**	-	E	-	*d*	D	-	E	-	-	-	Q	A	-	D	-	-	K	S	-	E	N	-	-	-	-	R	-	-	A	-
**ISR07(e)**	-	N	-	-	-	-	E	-	-	-	-	-	-	-	-	-	E	N	-	-	-	-	-	-	H	-	-	-	S	V
**E-391(e)**	-	Q	S		-	K	-	S	-	S	T	S	-	-	S	-	S	N	-	-	-	-	-	-	-	R	-	-	-	-
**MAD029(c)**	-	-	-	-	-	-	T	-	-	-	-	-	-	-	-	-	-	-	-	-	-	-	-	-	-	-	-	-	-	-
**NIE09(c)**	-	-	-	-	-	-	-	-	-	-	-	-	-	-	-	-	-	-	-	-	-	-	-	-	-	-	-	-	M	-
**EGY42(w)**	-	-	-	-	-	K	-	-	-	-	-	-	-	-	-	-	-	-	-	-	-	-	N	-	-	-	-	N	A	-
**EGY52(w)**	-	-	-	-	-	K	-	-	-	-	-	S	-	-	S	-	-	-	-	-	-	S	-	-	-	-	-	N	A	-
**VEN59(w)**	A	-	-	-	-	K	-	-	-	T	-	-	-	-	-	-	-	-	-	-	-	S	-	-	-	-	-	-	A	-
**MOR78(w)**	-	-	-	-	-	K	-	-	-	-	-	-	-	-	-	-	-	-	-	-	-	-	-	-	-	-	E	N	A	-
**KUW80(w)**	-	-	-	-	-	K	-	-	-	-	-	-	-	-	S	-	-	-	-	-	N	-	-	-	-	-	-	N	A	-

^a^ iVDPVs (i), cVDPVs (c), VDPVs found in sewage (e) and wild polioviruses (w) are shown. All these viruses were analysed in Fig 1 where EMBL Data Library accession numbers for published capsid sequences are shown. Names for some viruses are shortened in the table.

^b^ Amino acid in single letter code,—indicates the same amino acid as Sabin 2, *d* indicates deletion.

**Fig 3:**
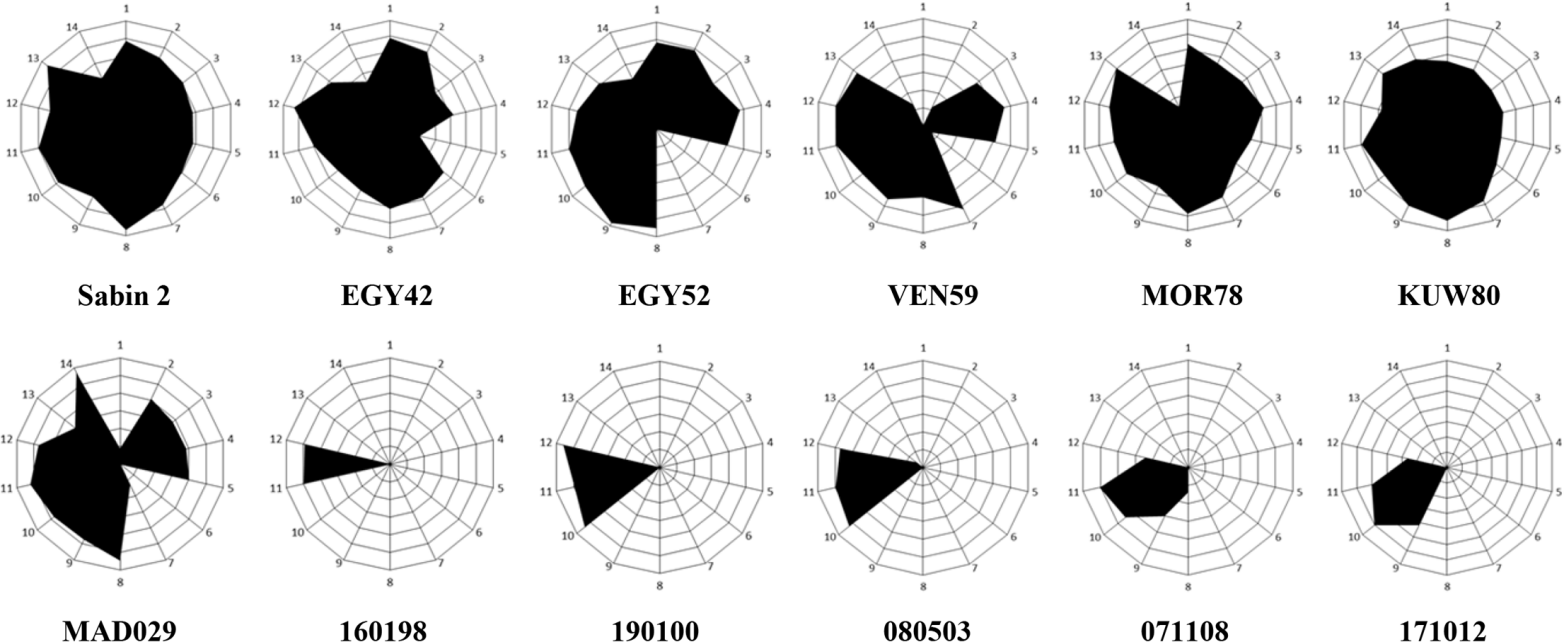
Antigenic structure of iVDPV strains. Radial diagrams representing the reactivity of poliovirus strains with Sabin 2-specific monoclonal antibodies. The results are shown as OD values at 492nm obtained in ELISA assays and expressed as normalised values relative to those obtained with antibody 1102 which reacted with all poliovirus strains. These values denote the average of two duplicate assays. Monoclonal antibodies used in the assay in the order 1 to 14 shown in the graph (from the top and clockwise), with antigenic site specificity shown in brackets, were: 969 (site 1), 435 (1), 433 (1), 434 (1), 436 (1), 1231 (2a), 1247 (2a), 1269 (2a), 1037 (2b), 1050 (3a), 1102 (3b), 1103 (3b), 1121 (3b) and 1051 (3b). Sabin 2 vaccine virus, iVDPV isolates from the patient (160198, 190100, 080503, 071108 and 171012), cVDPV strain MAD029 and wild strains (EGY42, EGY52, VEN59, MOR78 and KUW80) were used in the assays.

As a consequence, the iVDPV strains did not react at all with monoclonal antibodies against most of the known neutralising antibody sites (Fig 3). It was of interest that all isolates tested did react with antibodies specific for antigenic site 3b (1102 and 1103). In contrast, the wild polioviruses strains analysed, which span almost four decades in time and which were isolated in geographically distant locations, exhibited an antigenic structure much closer to that of Sabin 2 virus, reacting with at least one monoclonal antibody specific for each antigenic site (Fig 3). A cVDPV strain from Madagascar [21] also reacted with most monoclonal antibodies.

There was no evidence of sequences derived from Sabin 1 or Sabin 3 poliovirus vaccine strains nor sequences derived from other polio or non-polio human enterovirus isolates in any iVDPV genome examined. There was therefore no indication of recombination with other enteroviruses, although recombination within the iVDPV population is quite possible [22,23]. In contrast, virtually all cVDPV and wild type polio strains are recombinants with other group C enteroviruses and include sequences from the 5’NCR and/or the non-structural coding region [21,24].

**Fig 4:**
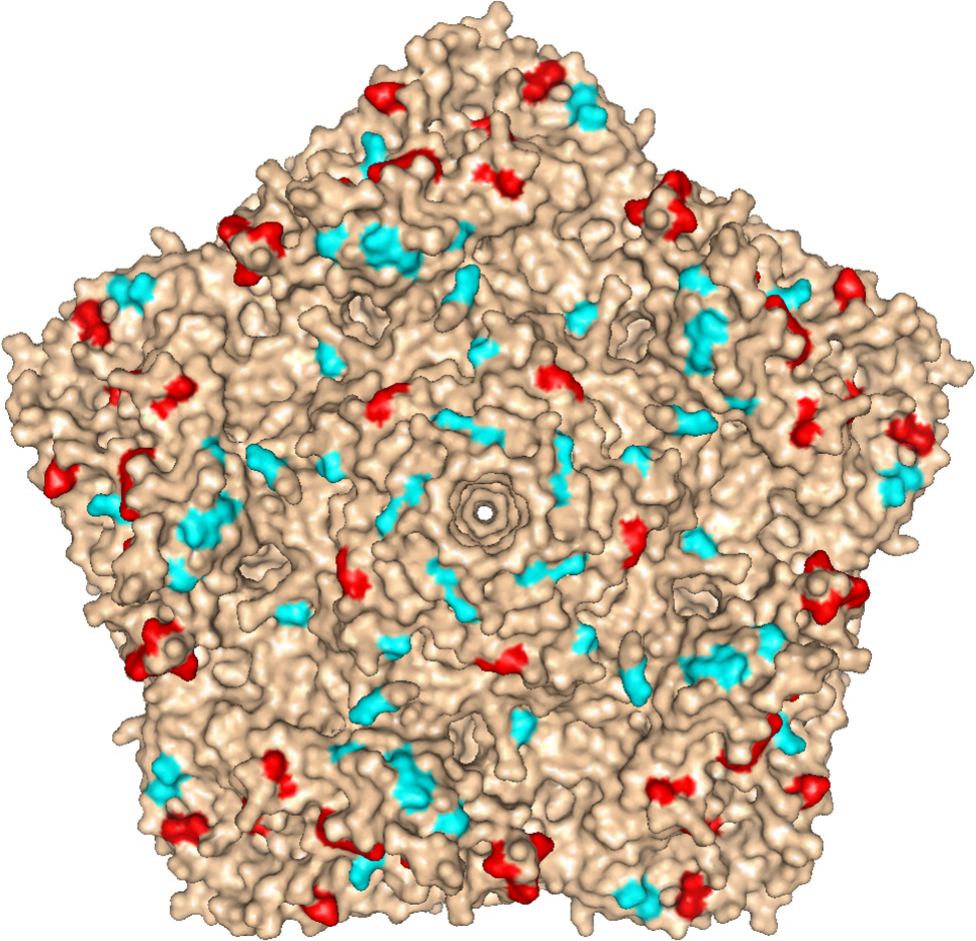
Location of mutations in iVDPV isolate 160198. Molecular surface diagram of the three-dimensional structure of type 2 wild poliovirus strain Lansing viewed from the outside of the virion [25]. A pentameric unit is represented. The virus particle consists of 60 protomers. Each protomer contains a single copy of VP1, VP2, VP3, and VP4 arranged in icosahedral symmetry. The location of mutations found in known antigenic sites of iVDPV isolate 160198 with respect to Sabin 2 vaccine strain are shown in red, other amino acid changes from Sabin 2 are displayed in cyan. The image was generated using PyMOL Molecular Graphics System, Version 1.7.0.3 software (Schrödinger, LLC).

**Fig 5:**
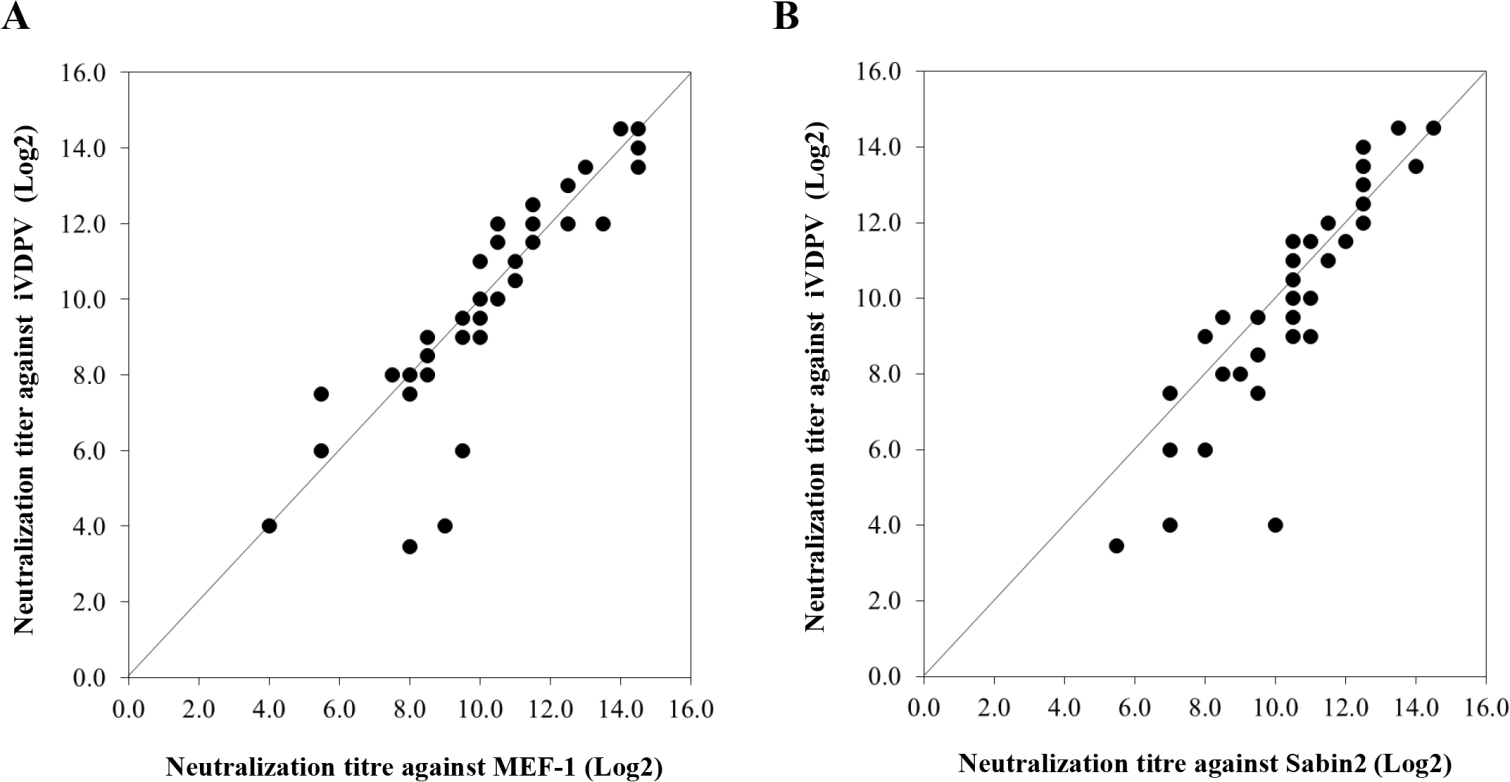
Neutralization of poliovirus strains by sera from fully immunised humans. The graphs represent comparison of neutralization titres in 40 sera from UK adults against iVDPV isolate 160198 versus MEF-1 (A) or versus Sabin 2 (B) vaccine strains in cell culture assays. The values are expressed as reciprocals (Log2) of the highest dilution of serum that protected 50% of the cell cultures determined by the Karber formula.

Despite the extensive antigenic changes found in iVDPV strains (Fig 4), human sera readily neutralised iVDPV isolate 160198, the most antigenically divergent strain (Fig 5). This isolate was the only one obtained by plaque purification so it might represent a minor variant with slightly different antigenic makeup.

The log2 geometric mean titre (GMT) of antibodies neutralizing iVDPV virus 160198 in 40 serum samples from UK adults was 9.96±2.78 comparable to that against MEF-1 (log2 GMT = 10.20±2.40), the wild poliovirus strain used for IPV production, and Sabin 2 (log2 GMT = 10.49±2.02), used for OPV production. These differences were not statistically significant (P = 0.95 for iVDPV vs MEF-1 and P = 0.36 for iVDPV vs Sabin2, for paired results using the Wilcoxon signed-rank test). The results suggest that antibodies to antigenic site 3b in human sera, partially conserved in iVDPV strains, may be sufficient to neutralise the virus. Alternatively, other conserved antigenic epitopes not detected by our murine antibody panel but present in the iVDPV strains, could have contributed to the high neutralization levels shown in human sera. These results are reassuring in that they indicate that vaccinated humans are well protected against infection with these highly drifted iVDPV strains. However, the sera tested here correspond to a selected group of UK healthy adults between 28–65 years of age who had been vaccinated with a full course of four OPV doses plus at least one dose of IPV. The UK switched from OPV to IPV for polio immunisation in 2004 so it would be helpful to test sera from cohorts that have only received IPV immunisation. Israel, which also switched from OPV to IPV at a similar date (2005), has recently detected the widespread circulation of type 1 wild poliovirus through environmental surveillance. There were no paralytic cases but, like the iVDPVs reported here, isolates from Israel showed antigenic differences from the corresponding vaccine strain which may have contributed to their ability to circulate in the context of IPV immunity [26].

**Fig 6:**
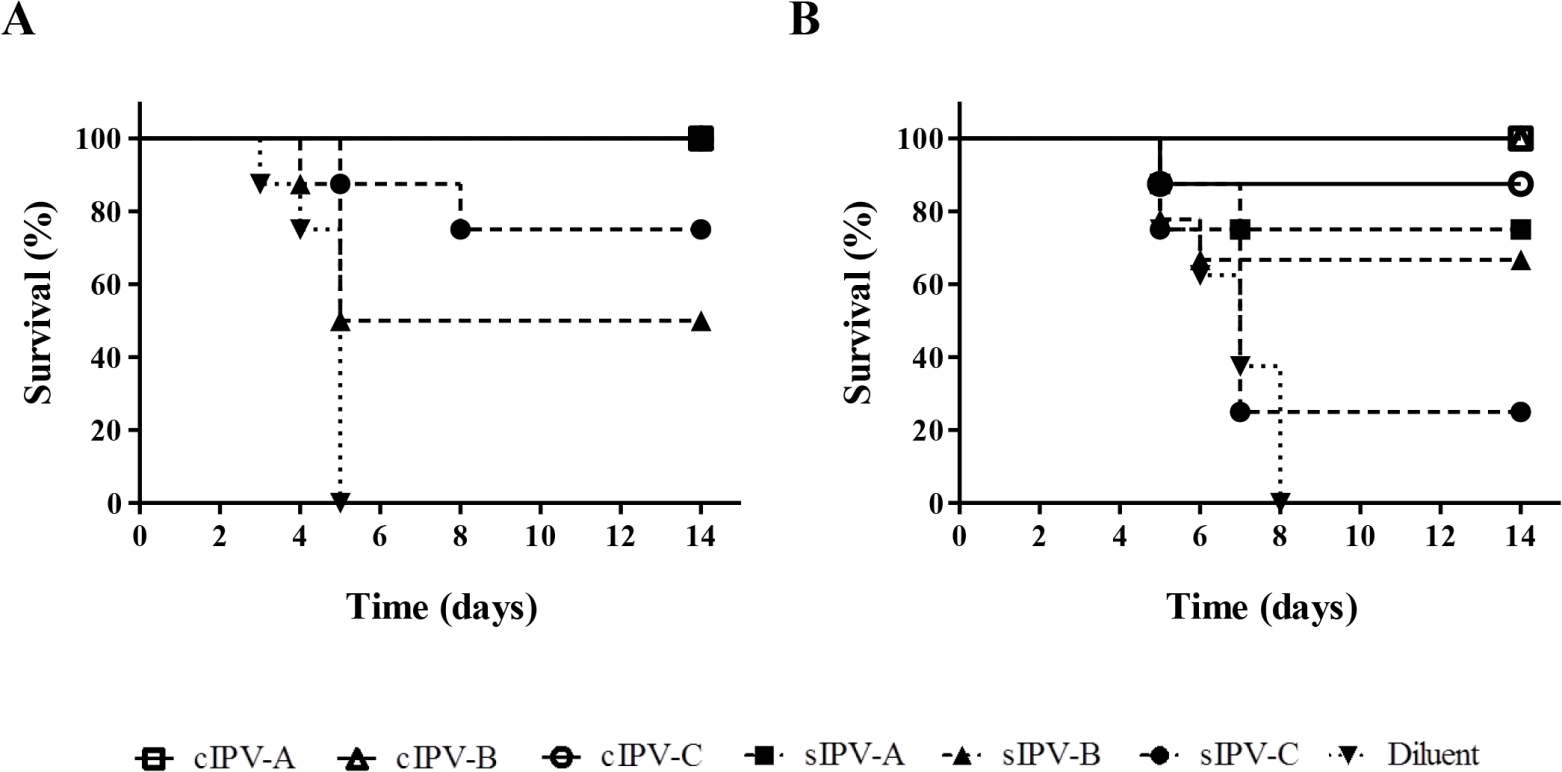
Survival curve analysis of Tg21-bx mice immunised with IPV and challenged with paralytic doses of poliovirus. Survival curves showing animals protected against paralysis caused by MEF-1 (A) or iVDPV strain 171012 (B) following immunisation with conventional (cIPV) products are shown with opened symbols and continuous lines; results for Sabin IPV (sIPV) products are shown as dashed lines and closed symbols; and survival data for mice injected with diluent (control) are shown as dotted lines and inverted triangles. All three cIPV products showed 100% protection against challenge with both viruses with the exception of cIPV-C that protected 7 out of the 8 animals used in the test. sIPV-A vaccine also protected all immunised animals against challenge with the MEF-1 strain.

We used a transgenic mouse model [27] to test the ability of different IPV products to protect against paralysis caused by iVDPV strains. Both conventional IPV (cIPV) based on wild poliovirus strains and Sabin IPV (sIPV) based on OPV strains were used (Fig 6).

All unimmunised control mice were severely paralysed by 5 days (MEF-1 virus) or 8 days (iVDPV strain) post-challenge. All three cIPV products protected against both challenge strains with only one animal developing paralysis. In contrast, sIPV products were less effective at protecting mice and showed variable responses between them. For all vaccines, the *in vitro* neutralizing antibody titers in sera from immunized mice were at least 7-fold lower against the iVDPV strain than they were against MEF-1 virus (Table 2).

**Table 2:** Neutralization titre against poliovirus in sera from transgenic mice immunised with IPV.^a^

	Virus strain	
IPV product	MEF-1	171012
sIPV-A	6.29±0.15	<3.00
sIPV-B	6.79±0.10	<3.00
sIPV-C	8.55±0.00	4.50±0.50
cIPV-A	7.79±0.10	5.00±0.00
cIPV-B	9.00±0.00	5.50±0.50
cIPV-C	8.58±0.50	5.29±0.29
Diluent	<3.00	<3.00

^a^The values are expressed as reciprocals (Log2) of the highest dilution of serum that protected 50% of the cell cultures determined by the Karber formula.

Our results highlight the need for improving the standardisation of sIPV products in terms of measuring vaccine potency and defining the protective human dose. The lower immunogenicity shown by type 2 sIPVs has been reported before [28,29].

In conclusion, we describe a patient who has been excreting highly virulent and antigenically modified type 2 poliovirus at high titres for a period estimated to be twenty eight years so far. This is by far the longest reported poliovirus excretion and represents the most comprehensive collection of iVDPV sequential isolates available. Provided antibody titres and immunisation coverage are maintained it is likely that the population will be protected against paralytic disease, but it is also possible that this virus could circulate in populations only using IPV as described in Israel for wild poliovirus [26], thus representing a possible source of polio re-emergence, particularly as these iVDPV strains are antigenically atypical and drifted from both Sabin 2 and MEF-1 vaccine strains. This is particularly relevant at present as there are imminent plans to remove type 2 poliovirus from OPV [15]. Moreover the use of IPV based on the Sabin strains is being encouraged by WHO for reasons of environmental safety and the data presented here suggest that it is less effective.

Of the total of 73 iVDPV cases that have been described between 1962 and 2014 [14], only seven of them involved infections lasting more than five years. The case described here represents the only individual of those seven known to be excreting at present. However, several highly drifted VDPV strains have recently been isolated from sewage samples in Slovakia, Finland, Estonia and Israel [30]. They included examples of all three poliovirus serotypes, although type 2 VDPVs were the most prevalent among them. These VDPV isolates showed molecular properties typical of iVDPVs described above indicating that an unknown number of these chronic excreters exist elsewhere. Interestingly, highly evolved type 1 and 2 VDPVs, which have been repeatedly isolated in Israel’s sewage in the last few years, do not appear to have spread widely as type 1 wild poliovirus did. Enhanced surveillance including sewage sampling and stool surveys should continue for as long as possible to search for the presence of iVDPV strains. The availability of efficient anti-viral treatments to interrupt virus replication in these individuals, actively being pursued at present [31], would also be vital as previous attempts have failed [18]. These measures are needed to be able to identify and manage the possible risks of iVDPV strains spreading and causing disease in patients and the general population, particularly in the light of changes in vaccination strategies as part of the polio eradication endgame and the absence of an established outbreak response strategy. Just as the use of new monovalent and bivalent vaccines proved essential to the elimination of wild poliovirus [32,33], novel vaccines unable to cause poliomyelitis would be useful at this stage of polio eradication. New polio vaccines such as those based on non-infectious virus-like particles or even new genetically designed stable live-attenuated versions [34–37] with no associated risk of producing VDPVs, might be required to resolve the “OPV paradox” that derives from using OPV to respond to outbreaks and generating new VDPVs as a consequence.

## Materials and Methods

### Case report

The patient is a white male from the UK. He received a full course of childhood immunisations, including OVP at 5, 7, and 12 months, with a booster at about 7 years of age. He was later diagnosed with common variable immunodeficiency (CVID) and started on intramuscular immunoglobulin therapy, which was changed to intravenous immunoglobulin after that [18].

### Poliovirus isolation from stool samples

Poliovirus was isolated from 10% stool suspensions using HEp-2c cells. Type 2 iVDPV strains 6735 and 04–44149261, isolated from two other immunodeficient patients in the UK, and type 2 wild poliovirus strains EGY42 (MEF-1 strain used for IPV production), EGY52, VEN59, MOR78 and KUW80 isolated from paralytic cases in 1942, 1952, 1959, 1978 and 1980, respectively, were also characterised in this study.

### Nucleotide sequencing of poliovirus genomes

#### VP1 gene

Purified viral RT-PCR DNA products were sequenced by the Sanger method on an ABI Prism DNA 377 Sequencer as specified by the manufacturer. Primers VDPV-F2 (5’-AGG GTT GTT GTC CCG YTG TCC AC-3’) and VDPV-R1 (5’-TAC ACA GCT GGY TAC AAA ATT TGC A-3’) were used to amplify and sequence VP1 gene sequences.

#### Full genome

Nearly full genomes of selected poliovirus isolates were sequenced using a deep sequencing method described before [38]. Sequence-independent amplification was performed to generate dsDNA templates using primers RA10-N8 (5´- GAC CAT CTA GCG ACC TCC CAN NNN NNN N -3 and RA10 (5´- GAC CAT CTA GCG ACC TCC CA -3´). Sequencing libraries were prepared using Nextera XT reagents and sequenced on a MiSeq using a 2 x 251 paired-end v2 Flow Cell and manufacturer’s protocols (Illumina). Raw sequence data were imported into Geneious R7 (Biomatters) and paired end reads combined. Data were filtered and aligned using a custom workflow with the following parameters: shotgun primer RA10 and Nextera adaptor/index sequences were trimmed from 5 and 3´ ends with a minimum 5 bp overlap; reads were trimmed to have an average error rate < 1%, no bases with a quality of < Q30 and no ambiguities. Reads were then mapped to the sequence of the Sabin 2 poliovirus reference (GenBank AY184220) and a consensus sequence obtained for each poliovirus strain. Nucleotide sequences of iVDPV and wild poliovirus isolates described in this paper have been deposited in the DDBJ/EMBL/GenBank and have been assigned accession numbers KR817050-KR817066.

#### Phylogenetic analysis

Poliovirus sequences were compared to those of other polioviruses in the DDBJ/EMBL/GenBank database. Representative related sequences were included in the phylogenetic analyses. Neighbour-joining phylogenetic analysis was performed with MEGA6 [39] using the maximum composite likelihood substitution model with gamma distributed substitution rates. In addition, a Bayesian Monte Carlo Markov Chain (MCMC) analysis of VP1 sequences (42 iVDPV isolates from the patient obtained throughout the period of virus excretion), as implemented in BEAST v1.8.1 [40], was used for the estimation of the rate of evolution and the date of the initiating OPV dose. The general time reversible (GTR) model of substitution with invariant sites was the best-fitting model of evolution as estimated by jMODELTEST [41]. Two independent chains of 10 million steps each were run under the strict clock model, assuming a constant substitution rate as estimated from the data set. The samples’ collection dates were included as temporal data. Effective sample size values were monitored for consistency using Tracer v1.6.

### Neurovirulence in transgenic mice

Tg21-Bx transgenic mice expressing the human poliovirus receptor were inoculated intramuscularly (left hind limb) with 50 μl of 10-fold viral dilutions and daily clinical scores were recorded for 14 days. Eight mice were used for each viral dilution. The Probit method was used to calculate the 50% paralytic dose (PD_50_) and associated 95% confidence intervals for each poliovirus challenge [27].

### Antigenic characterization

The antigenic properties of poliovirus isolates were studied by analysing their ability to bind Sabin 2-specific monoclonal antibodies in ELISA assays using testing formats described before [42]. Antibodies corresponding to antigenic sites 1, 2a, 2b and 3b were used in these assays. Solutions containing equivalent concentrations of poliovirus measured as 50% cell culture infectious doses (CCID_50_) per ml were selected. The results represent the OD values at 492nm and were expressed as normalised values relative to those obtained with antibody 1102 which reacted with all poliovirus strains.

### Immunization/Challenge experiments in transgenic mice

Tg21-Bx mice (8 per test group) were immunised twice by intraperitoneal injection with IPV (using the equivalent of 1 human dose/mouse) or minimum essential medium (diluent control) at an interval of 2 weeks. Twenty-one days after the last dose, mice were challenged with the equivalent of 25 times the PD_50_ of live poliovirus and daily clinical scores were recorded for 14 days [27]. Vaccines used in these experiments were kindly donated by various manufacturers and were coded to maintain anonymity. This work was part of the characterisation of these vaccines as reference standards. It is important to note that given the high PD_50_ values observed for type 2 poliovirus strains in our transgenic mouse neurovirulence model, mice were challenged with very large amounts of virus (around 10^8^ CCID_50_/mouse).

### Titration of human and mouse sera for poliovirus neutralising antibodies

Neutralizing antibody titres in serum samples were determined by a standard microneutralization assay in 96-well plates. Two-fold serial dilutions of serum were preincubated with one hundred CCID_50_ of virus for 2 hours at 36°C. HEp-2C cells were added to each well, and survival at day 5 post-infection determined by staining with a 0.1% Naphthalene black solution. Antibody titres were expressed as reciprocals (Log2) of the highest dilution of serum that protected 50% of the cell cultures determined by the Karber formula. Virus challenge doses were confirmed by back-titration. The significance of pairwise differences in neutralization titres of human sera against MEF-1, Sabin 2 and iVDPV 160198 strain was determined using the Wilcoxon signed-rank test.

### Ethics statement

The adult subject who provided stool samples gave written informed consent. Adults who provided blood samples also provided written informed consent. The work complies with the Caldicott Principles and Recommendations for patient confidentiality set up by the UK National Health Services (NHS). All links to personal details that could be used to identify individuals were removed and data were analysed anonymously when possible. No samples from children were involved in the study. The study was approved by NIBSC’s Ethics and Human Materials Advisory Committees. NIBSC’s Animal Welfare and Ethical Review Body approved the application for Procedure Project Licence Number 80/2478 which was approved by the UK Government Home Office and under which animal care and protocols shown in this paper were conducted. All animal care and protocols used at NIBSC adhere to UK regulations (Animals, scientific procedures, Act 1986 that regulates the use of animals for research in the UK) and to European Regulations (Directive 2010/63/Eu of the European Parliament on the protection of animals used for scientific purposes). The experiments in mice shown here were carried out following protocols 4 and 5 within Home Office Procedure Project Licence Number 80/2478 referred above.

## Supporting Information

S1 Table Amino acid changes in iVDPV isolates. Mutations in amino acid sites between iVDPV isolates and Sabin 2 vaccine virus are shown.
(DOCX)
