# Supplementary material for: Twenty-Eight Years of Poliovirus Replication in an Immunodeficient Individual: Impact on the Global Polio Eradication Initiative
Source: PLoS Pathog. 2015 Aug 27;11(8):e1005114. doi: 10.1371/journal.ppat.1005114 (PMC4552295; doi:10.1371/journal.ppat.1005114)
Supplement: S1 Table — Mutations in amino acid sites between iVDPV isolates and Sabin 2 vaccine virus are shown. (DOCX) [file ppat.1005114.s001.docx]

**Supporting information**

| **S1 Table. S1 Table. Amino acid changes in iVDPV isolates**. Mutations in amino acid sites between iVDPV isolates and Sabin 2 vaccine virus are shown. | | | | | | |
| --- | --- | --- | --- | --- | --- | --- |
|  | Poliovirus isolate | | | | | |
| Amino acid no.^a^ | Sabin 2 | 160198 | 190100 | 080503 | 071108 | 171012 |
| VP4-21 | G^b^ | - | - | D | D | D |
| VP4-42 | S | N | N | N | N | N |
| VP4-43 | K | - | R | R | R | - |
| VP2-45 | T | A | A | A | - | A |
| VP2-72 | R | N | N | N | N | N |
| VP2-137 | T | M | M | M | M | I |
| VP2-141 | F | Y | Y | Y | - | - |
| VP2-155 | E | V | V | V | V | V |
| VP2-157 | K | E | - | - | - | - |
| VP2-158 | G | R | - | - | - | - |
| VP2-164 | T | - | D | N | N | N |
| VP2-168 | N | - | - | S | - | S |
| VP2-172 | N | E | K | K | K | K |
| VP2-186 | V | A | A | A | A | A |
| VP2-207 | L | I | I | I | I | I |
| VP2-221 | T | A | - | - | - | - |
| VP2-240 | T | - | - | - | A | A |
| VP3-6 | N | - | D | D | D | D |
| VP3-19 | Y | H | - | - | - | - |
| VP3-41 | R | H | H | H | H | H |
| VP3-55 | L | F | F | F | F | F |
| VP3-61 | R | H | H | H | H | H |
| VP3-62 | K | R | R | R | R | R |
| VP3-73 | S | T | T | T | T | T |
| VP3-75 | T | A | A | A | A | A |
| VP3-78 | S | T | T | T | T | T |
| VP3-80 | T | E | N | N | N | D |
| VP3-85 | L | F | F | F | F | F |
| VP3-144 | S | - | N | N | N | K |
| VP3-158 | I | V | V | V | V | V |
| VP3-209 | D | V | V | V | V | V |
| VP3-234 | E | K | K | K | K | K |
| VP3-236 | M | I | I | - | I | I |

| **S1 Table contd.** | | | | | | |
| --- | --- | --- | --- | --- | --- | --- |
|  | Poliovirus isolate | | | | | |
| Amino acid no. | Sabin 2 | 160198 | 190100 | 080503 | 071108 | 171012 |
| VP1-3 | G | E | E | E | E | E |
| VP1-5 | M | I | I | I | I | I |
| VP1-8 | G | - | - | S | S | S |
| VP1-9 | A | V | V | - | - | - |
| VP1-10 | E | - | - | A | A | A |
| VP1-19 | V | T | T | N | N | N |
| VP1-21 | P | - | - | - | - | T |
| VP1-22 | T | - | A | A | A | A |
| VP1-24 | T | - | - | - | A | A |
| VP1-25 | N | G | G | S | - | - |
| VP1-26 | S | - | - | - | N | - |
| VP1-32 | P | - | S | S | S | S |
| VP1-69 | R | - | - | K | K | K |
| VP1-75 | S | T | T | T | T | T |
| VP1-77 | V | I | I | I | I | I |
| VP1-99 | K | N | N | N | N | N |
| VP1-101 | A | T | T | T | - | - |
| VP1-103 | R | K | K | K | K | K |
| VP1-143 | I | T | T | T | T | T |
| VP1-145 | A | S | S | S | S | S |
| VP1-171 | N | D | D | D | D | D |
| VP1-219 | G | S | - | - | - | - |
| VP1-220 | Q | H | H | H | H | H |
| VP1-221 | A | - | - | - | - | T |
| VP1-222 | S | A | A | A | A | A |
| VP1-223 | T | - | - | A | A | del |
| VP1-224 | E | D | D | D | D | D |
| VP1-235 | N | D | D | D | D | D |
| VP1-257 | I | V | V | V | V | V |
| VP1-261 | M | L | L | L | L | L |
| VP1-280 | F | Y | Y | Y | Y | Y |
| VP1-289 | G | E | E | E | E | E |

| **S1 Table contd.** | | | | | | |
| --- | --- | --- | --- | --- | --- | --- |
|  | Poliovirus isolate | | | | | |
| Amino acid no. | Sabin 2 | 160198 | 190100 | 080503 | 071108 | 171012 |
| 2A-6 | N | - | S | S | S | S |
| 2A-27 | L | F | F | F | F | F |
| 2A-32 | S | C | C | C | C | C |
| 2A-42 | A | T | T | T | T | T |
| 2A-47 | L | S | S | S | S | S |
| 2A-51 | S | - | - | T | T | T |
| 2A-56 | S | R | R | R | R | R |
| 2A-59 | T | A | - | - | A | A |
| 2A-67 | R | K | - | - | - | - |
| 2A-76 | I | V | V | V | V | V |
| 2B-22 | G | S | S | S | S | S |
| 2B-29 | T | A | A | A | - | - |
| 2B-30 | S | - | N | - | - | - |
| 2B-45 | I | V | V | V | V | V |
| 2B-82 | K | - | R | R | - | - |
| 2B-95 | I | V | - | - | - | - |
| 2B-96 | K | R | R | R | - | - |
| 2C-3 | S | G | G | G | G | G |
| 2C-75 | I | - | - | V | V | V |
| 2C-96 | H | L | L | L | L | L |
| 2C-112 | V | I | I | I | I | I |
| 2C-225 | S | N | - | - | - | N |
| 2C-246 | V | M | M | M | M | M |
| 2C-248 | I | - | - | - | - | M |
| 2C-255 | S | C | - | - | - | - |
| 2C-303 | I | - | - | - | V | V |
| 2C-311 | V | I | I | I | I | I |
| 3A-12 | V | - | - | I | I | I |
| 3A-39 | K | - | - | - | R | R |
| 3A-46 | I | - | - | - | V | V |
| 3B-8 | N | I | I | - | - | - |
| 3C-31 | Y | H | H | H | H | H |
| 3C-47 | I | - | - | - | V | V |
| 3C-48 | A | V | V | V | I | I |
| 3C-61 | A | S | S | S | S | S |
| 3C-78 | K | R | R | R | R | R |
| 3C-182 | I | T | T | N | D | D |
| 3D-6 | M | I | - | - | - | - |
| 3D-46 | N | - | - | S | S | - |
| 3D-51 | R | K | K | K | K | K |
| 3D-69 | D | E | E | E | E | E |
| 3D-142 | R | Q | Q | Q | Q | Q |
| 3D-204 | V | I | I | I | - | - |
| 3D-276 | K | - | R | - | - | - |
| 3D-311 | K | R | - | - | - | - |
| 3D-413 | H | - | - | - | I | - |
| ^a^Amino acid is indicated by the name of protein followed by residue number.  ^b^Amino acid in single letter code, - indicates the same amino acid as Sabin 2. | | | | | | |
